# El Niño and coral larval dispersal across the eastern Pacific marine barrier

**DOI:** 10.1038/ncomms12571

**Published:** 2016-08-23

**Authors:** S. Wood, I. B. Baums, C. B. Paris, A. Ridgwell, W. S. Kessler, E. J. Hendy

**Affiliations:** 1 School of Earth Sciences, University of Bristol, Queens Road, Bristol BS8 1RJ, UK; 2 Department of Biology, Pennsylvania State University, 208 Mueller Lab, University Park, State College, Pennsylvania 16802, USA; 3 Department of Ocean Sciences, Rosenstiel School of Marine and Atmospheric Science, University of Miami, Miami, Florida 33149-1098, USA; 4 Department of Earth Sciences, University of California, Riverside, California 92521, USA; 5 School of Geographical Sciences, University of Bristol, University Road, Bristol BS8 1SS, UK; 6 Pacific Marine Environmental Laboratory/NOAA, Seattle, Washington 98115, USA

## Abstract

More than 5,000 km separates the frequently disturbed coral reefs of the Eastern Tropical Pacific (ETP) from western sources of population replenishment. It has been hypothesized that El Niño events facilitate eastward dispersal across this East Pacific Barrier (EPB). Here we present a biophysical coral larval dispersal model driven by 14.5 years of high-resolution surface ocean current data including the extreme 1997–1998 El Niño. We find no eastward cross-EPB connections over this period, which implies that ETP coral populations decimated by the 1998 bleaching event can only have recovered from eastern Pacific sources, in congruence with genetic data. Instead, rare connections between eastern and central Pacific reefs are simulated in a westward direction. Significant complexity and variability in the surface flows transporting larvae mean that generalized upper-ocean circulation patterns are poor descriptors of inter-regional connectivity, complicating the assessment of how climate change will impact coral gene flow Pacific wide.

## Abstract (web summary)

Over 5,000 km of open ocean separate central and eastern Pacific coral reefs. Here, the authors combine a biophysical dispersal model with genetic data to show that eastern Pacific coral populations have been isolated from western sources of larval recruitment since the 1997-98 El Niño-induced bleaching event.

## Graphical abstract

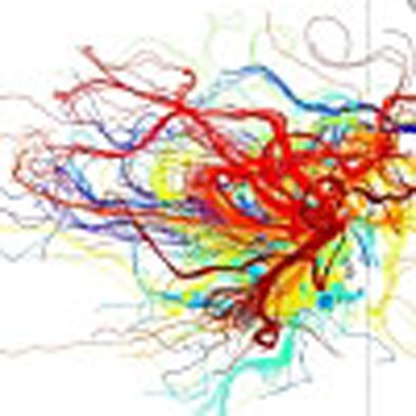

Coral populations of the Eastern Tropical Pacific (ETP) survive in some of the harshest conditions for reef development worldwide^1^. Limited shallow-water habitat, combined with frequent environmental disturbances and upwelling of cool, low pH waters that restrict skeletal calcium carbonate production results in small, patchy reefs prone to erosion^2^,^3^,^4^. Consequently, ETP reefs are considered modern-day analogues for future reefs under rising atmospheric CO_2_ (ref. 2), although cool upwelled waters may also buffer twenty-first century ETP reefs from the rising sea surface temperatures expected to devastate western Pacific populations^5^. This environmental setting makes ETP reefs an interesting case study, both as vulnerable marginal populations and as potential refuges for coral reef biodiversity under climate change.

The El Niño-Southern Oscillation (ENSO) dominates climatic variability across the tropical Pacific. Warm El Niño or cold La Niña phases, occurring every 2–7 years, cause wide-scale ecological disturbances in the region^3^. In particular, anomalously high temperatures associated with El Niño induce ‘coral bleaching'—the expulsion of symbiotic photosynthetic algae that provide corals with the energy for reef formation—in severe cases leading to the death of the host coral^6^. The extreme El Niño events of 1982–1983 and 1997–1998, considered the most intense of the past century^7^,^8^, caused widespread severe bleaching, followed by coral mortality and local extinctions within the eastern Pacific^9^,^10^,^11^,^12^. While some reefs have since recovered^13^,^14^,^15^, others have not^16^, and East Pacific reef recovery is notably slower than other regions globally^17^.

The delayed recovery of ETP reefs can be attributed, in part, to their extreme geographic isolation and therefore limited sources of population replenishment^17^. Broadcast spawning corals, in common with many benthic marine species, disperse via a pelagic larval life history stage that drifts with ocean currents over potentially vast distances^18^. However, barriers to dispersal exist where the oceanographic distance between habitats, driven by the speed and direction of the connecting ocean currents, exceeds larval lifetimes of the species in question. More than 5,000 km of open ocean, the East Pacific Barrier (EPB), separates eastern Pacific coral populations from their western counterparts, a distance historically considered impassable for many marine larvae^3^,^19^,^20^ (Fig. 1). Indeed, recent population genetic data for the trans-Pacific coral *Porites lobata* suggest that eastern Pacific populations have been isolated from those of the central Pacific for at least several generations^21^ (considering the multi-century lifespans of massive poritid colonies^15^ this equates to potentially thousands of years of separation). It follows that recovery of ETP reefs following disturbance may be highly reliant on survivors of affected populations^14^,^17^ rather than being fuelled by more diverse central Pacific populations.

However, the presence of a number of trans-Pacific species in the ETP, such as *P. lobata* and *Pocillopora damicornis*, implies that the EPB has, at least historically, been breached^3^. When and how frequently cross-EPB genetic exchange occurs has implications for both the origin of ETP reef faunas^3^,^19^,^22^ and their resilience to continuing pressures such as warming and ocean acidification. Prevailing upper-ocean flow across the tropical Pacific is westward via the North and South Equatorial Currents (NEC/SEC; Fig. 1). However, inferred transport times across the EPB exceed the larval life span of most marine species^19^,^20^,^23^. Instead, it has commonly been proposed that El Niño events may facilitate cross-Pacific dispersal of marine larvae counter to the prevailing flow, via enhanced flow of the eastward North Equatorial Counter-Current (NECC; Fig. 1)^3^,^14^,^19^,^20^,^23^,^24^,^25^,^26^,^27^. Because future climate change may potentially modulate the strength, frequency and ‘type' of El Niño events^28^,^29^, and thereby coral reef disturbance, connectivity and recovery rates, it is important to explore whether this hypothesis is correct.

It is impossible to measure larval dispersal directly over large distances. Instead, in this paper, we test whether eastward dispersal across the EPB is more likely during El Niño events using a biophysical model of the dispersive larval stage of corals in the surface ocean. We model the oceanographic transport of larvae of a generic, ubiquitously distributed broadcast spawning coral with high dispersal potential over 14.5 years (1997–2011), covering a full spectrum of ENSO conditions, including the extreme El Niño of 1997–1998 (Fig. 2a), as well as other sources of variability in surface circulation. Despite maximizing the potential for long-distance dispersal in the model, no connections from the central Pacific to any eastern Pacific reefs are obtained for any of the 5.14 billion larval paths modelled over the entire study period. This implies that eastern Pacific broadcast spawning coral populations have been isolated from central Pacific sources of larval recruitment since at least 1997, a finding corroborated by genetic data for the widespread Pacific coral *P. lobata*^21^. Instead, the EPB is breached in the opposite direction by larvae released from both the Galapagos archipelago and Clipperton Island in the model. However, whether these westward connections could be realised will depend on the timing of coral spawning and the negative reproductive impact of El Niño-linked temperature stress.

## Results

### Modelled potential larval connectivity

**Figure 1:**
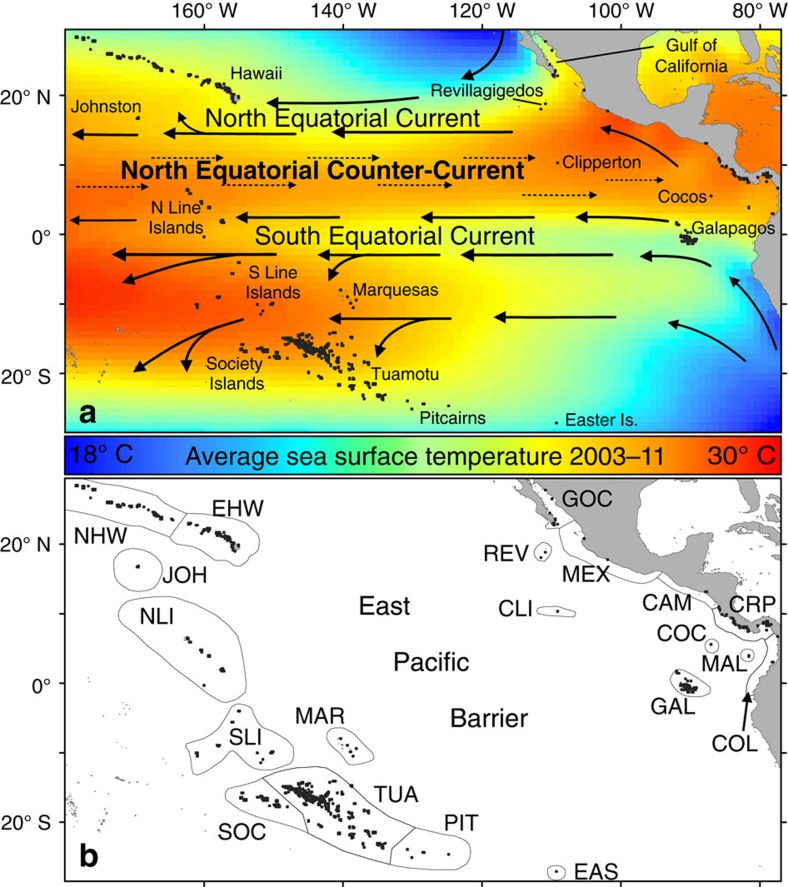
Model domain. (**a**) Central and eastern Pacific model reef cells (black squares), observed sea surface temperatures (average of HadISST monthly means for the period 2003–2011) and schematic of large-scale surface currents (black arrows). (**b**) Regional reef delimitations (grey outlines, adapted from multispecies coral ‘ecoregions'^30^) and codes used throughout the manuscript; CAM, Pacific Central America (Guatemala, El Salvador, Honduras, Nicaragua); CRP, Pacific Costa Rica and Panama; COL, Columbia and Ecuador; COC, Cocos Island; CLI, Clipperton Island; EHW, Eastern Hawaiian Islands; GAL, Galapagos Islands; GOC, Gulf of California; JOH, Johnston Atoll; MAR, Marquesas Islands; MAL, Malpelo Island; MEX, Pacific Mexico; NHW, northern Hawaiian Islands; EAS, Easter Island; NLI, northern Line Islands; PIT, Pitcairn Islands; REV, Revillagigedo Islands; SLI, Southern Line Islands; SOC, Society Islands; TUA, Tuamoto Archipelago.

We released 1,600 larvae daily from each of 636 reef locations in the model, grouped into 10 eastern and 10 central Pacific ‘ecoregions'^30^ (Fig. 1b), over a total of 14.5 years (1 January 1997 to 3 July 2011). Model larvae were advected with high-resolution, eddy-resolving ocean current reanalysis data for a maximum of 120 days—the maximum competency duration reported for any broadcast spawning coral species^31^—and subjected to a daily probability of mortality to mimic inter-individual variability in survival durations^31^ (see Methods). The number of model larvae successfully ‘settling' (defined as arrival within a certain distance of suitable habitat at one of the 636 reef sites, see Methods) over the entire 14.5-year study period are plotted as a connectivity matrix (Fig. 3a), which defines potential connectivity between regions (Fig. 1b).

**Figure 2:**
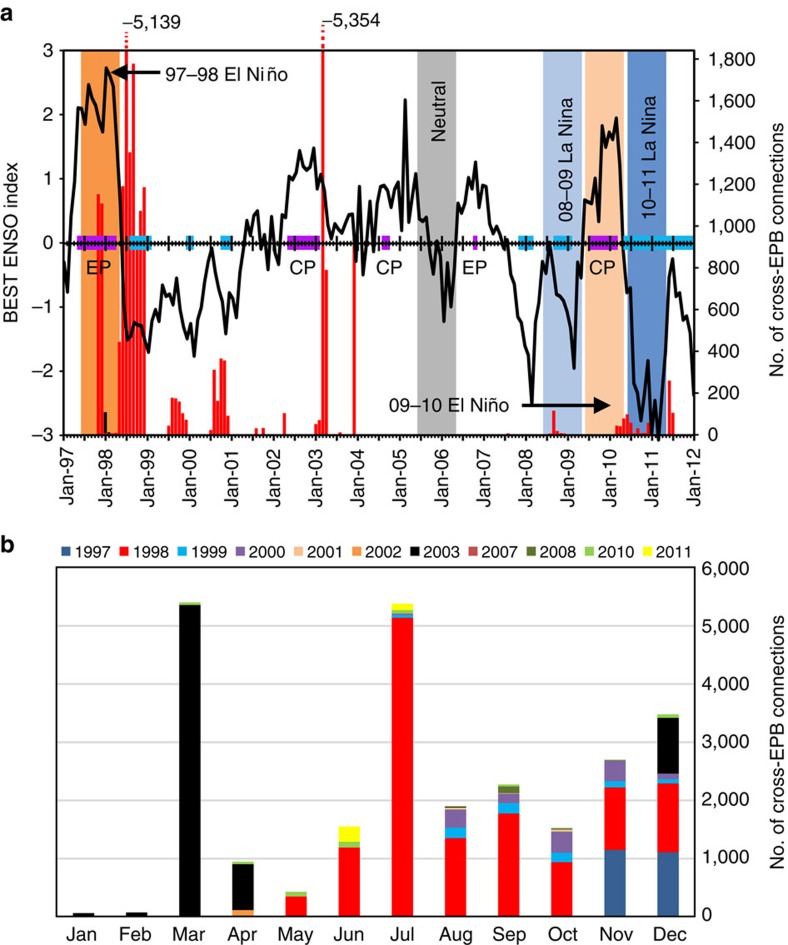
Cross-EPB connections. (**a**) The number of successful cross-EPB connections (right axis) westward from the ETP to the central Pacific over the model period (releases from 1 January 1997 to 3 July 2011) shown by month of release (*x* axis) from the Galapagos (GAL; red vertical bars) and Clipperton (CLI; black bars). The thick black line shows corresponding monthly ENSO phase and strength (Bivariate EnSo Timeseries ‘BEST'^69^, left axis). El Niño (purple; identified by type as eastern Pacific, ‘EP' or central Pacific, ‘CP'^32^,^70^) and La Niña (blue) events are marked along the central axis (as defined by the BEST index, relaxed criteria; www.esrl.noaa.gov/psd/people/cathy.smith/best/table33.txt). The representative annual periods (June through May) used in the comparison of dispersal variability under different ENSO conditions are highlighted as vertical bars; 1997–1998 (dark orange) and 2009–2010 (light orange) El Niño, 2008–2009 (light blue) and 2010–2011 (dark blue) La Niña and 2005–2006 neutral period (grey). (**b**) Number of successful cross-EPB connections from the Galapagos only by release month for the 11 years over which such connections occurred.

**Figure 3:**
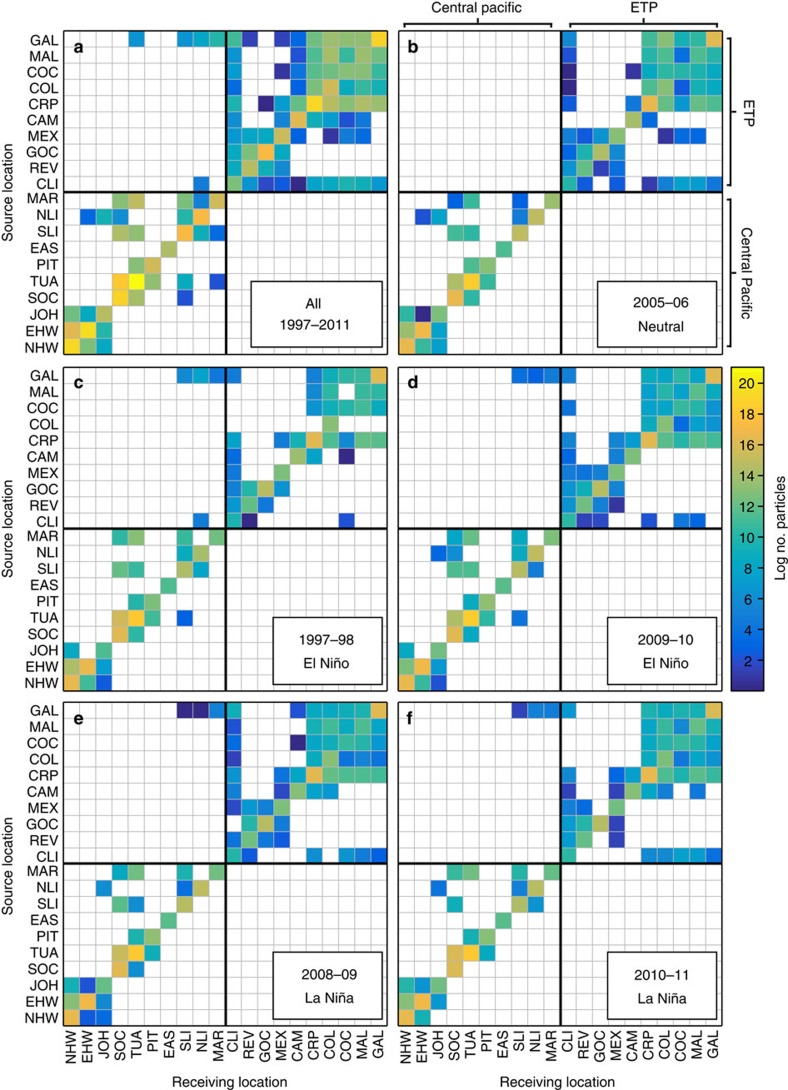
Potential connectivity matrices. Connectivity matrices showing the number of model larvae exchanged between each release (*y* axis) and receiving (*x* axis) region for (**a**) the entire study period (releases from 1 January 1997 to 3 July 2011), and representative annual periods (releases from 1 June to 31 May) for (**b**) the 2005–2006 neutral period, (**c**) 1997–1998 El Niño, (**d**) 2009–2010 El Niño, (**e**) 2008–2009 La Niña and (**f**) 2010–2011 La Niña (shown in Fig. 2a). Region delimitations used in the matrices are given in Fig. 1b. The central and eastern tropical Pacific (ETP) regions have been divided by a thick black line, representing the eastern Pacific barrier (EPB).

No eastward dispersal across the EPB was observed at any point over the entire study period (Fig. 3a). Contrary to the hypothesis of increased eastward cross-EPB dispersal during El Niño, rare connections instead occurred in the opposite direction at various points during the study period (Figs 2 and 3); a southerly route from the Galapagos (GAL) to the Marquesas (MAR), Line Islands (NLI/SLI) and Tuamotu (TUA) for releases during 11 of the 14.5 years modelled, in particular mid-1998 and early-2003 (Fig. 2b; taking >77 days) and a northerly route from Clipperton (CLI) to the Northern Line Islands (NLI) for larval releases during just 2 months (January–February) of the 1997–1998 El Niño (taking >104 days).

### Variability in potential connectivity outcomes

We also explore inter-annual variability in the potential connectivity output occurring under different ENSO conditions (Fig. 2a) by extracting the connectivity data for five representative annual periods (Fig. 3b-f): the extreme 1997–1998 El Niño (an eastern Pacific event^32^), a weaker, central Pacific El Niño (2009–2010), the extreme 2010–2011 La Niña, a weaker La Niña (2008–2009) and a neutral period (2005–2006). As El Niño/La Niña events usually peak around December, releases for the period 1 June through 31 May were selected to encompass a full year of seasonal variability including both the build-up and decline of these events.

Broad-scale patterns of regional-scale connectivity, such as the isolation of the ETP and Easter Island from the central Pacific and relative isolation of Hawaii from the central Pacific and the northern (north of Honduras; central American region; Fig. 1b) from the southern ETP, were persistent between years (Fig. 3). However, inter-regional connectivity within the eastern and central Pacific regions was sensitive to stochastic, daily to inter-annual variability as evident by changes in connections between matrices (Fig. 3). For example, connections within the southern ETP from the remote Columbia/Ecuador (COL) region north to Costa Rica/Panama (CRP), Cocos (COC) and Malpelo (MAL) Islands, and west to the GAL were obtained in the 2009–2010 central Pacific El Niño, both the 2008–2009/2010–2011 La Niñas and the 2005–2006 neutral period, but not the stronger 1997–1998 eastern Pacific El Niño, whereas a connection from the central American (CAM) to CRP region was obtained in both La Niñas, the 1997–1998 El Niño and the neutral period but not the weaker 2009–2010 central Pacific El Niño.

### Biophysical model sensitivity

While we parameterize the model to maximize the potential for long-distance dispersal (for example, applying the maximum larval competency duration values reported for any broadcast spawning coral in the literature and releasing model larvae year-round, see Methods), the ability to capture rare dispersal events may be affected by the biological parameters used. To test whether the result of solely westward dispersal across the EPB in the model is sensitive to model parameter choice, we further enhanced the potential for long-distance dispersal over the strongest El Niño period in the model (June 1997 to May 1998), as the most likely period for eastward cross-EPB dispersal, by extending the larval duration and reducing the mortality rate (see Methods). Both changes increased inter-regional connectivity predominantly within the ETP and also to a lesser extent central Pacific regions; however, the outcome of no eastward connections across the EPB remained unchanged (Supplementary Fig. 1).

**Figure 4:**
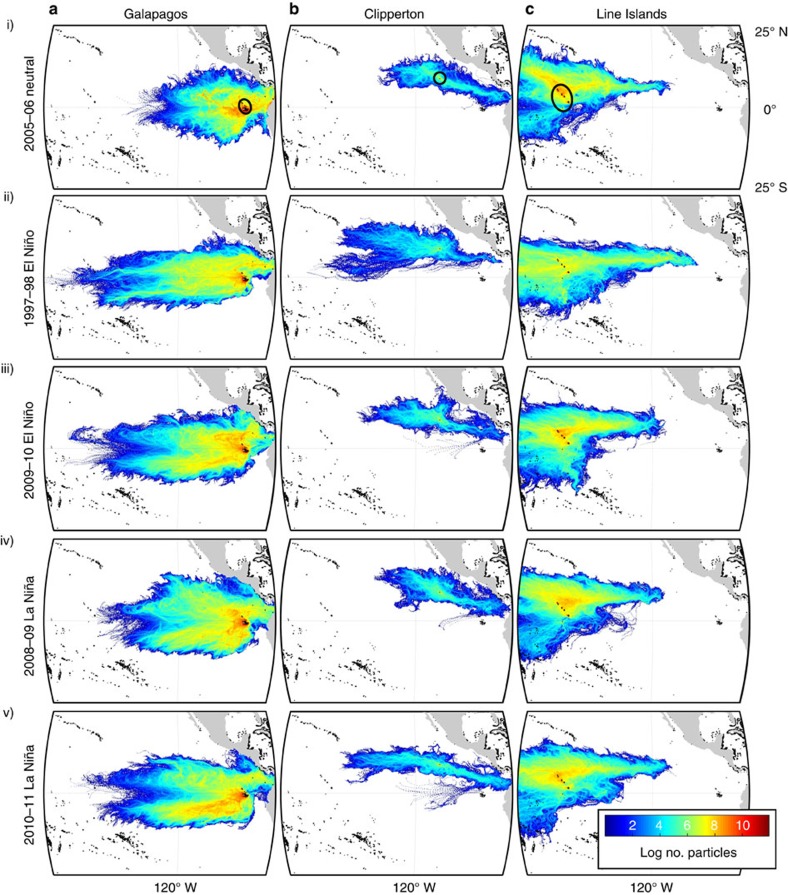
‘Oceanography only' larval paths. Modelled dispersal paths conducted with a larval duration of 120 days, excluding mortality or settlement. Twenty larvae were released from each habitat cell per day for a full annual period (1 June to 31 May), from (**a**) the Galapagos (43 release cells), (**b**) Clipperton (1 cell) and (**c**) Northern Line Islands (22 cells) over (i) the 2005–2006 neutral period, (ii) 1997–1998 eastern Pacific El Niño, (iii) 2009–2010 central Pacific El Niño, (iv) 2008–2009 La Niña and (v) 2010–2011 La Niña (highlighted in Fig. 2a). Paths are plotted as larval densities, calculated as the number of particles passing through each cell on a 1/6° grid. Animations of these plots by release month can be viewed in Supplementary Movies 1 and 2 (Northern Line Islands), Supplementary Movies 3 and 4 (Galapagos) and Supplementary Movies 5 and 6 (Clipperton).

We also ran the model for the five representative annual periods detailed above (with reduced larval numbers due to computational constraints) excluding larval biology (mortality and settlement; that is, advecting all larvae for the full 120 days), for releases from the GAL (Fig. 4a), CLI (Fig. 4b) and the NLI (Fig. 4c) as the most likely sources of dispersal across the EPB. Plotted larval trajectories from this model run show that although a few eastward larval trajectories from the NLI reach the longitude of CLI within 120 days (during the 1997–1998 El Niño period; Fig. 4c,ii), they pass to the south of the island by ∼1–2° (∼100–200 km).

### Comparison of the biophysical model with genetic data

The model was not parameterized to represent a specific coral species—an aim that would be impossible given issues such as limited temporal and spatial data on population sizes, timing of spawning, larval characteristics and recent recognition of the presence of cryptic species in the region. Instead, a species trait approach has been taken, with the model parameterized to represent a generic, broadcast spawning coral with high dispersal potential. However, to explore how far the model results may be applicable for a specific species, and identify differences where research effort should be focused (in terms of modelling, biological observations and sensitive locations for additional genetic sampling), we compare connections generated by the biophysical model with recently published genetic differentiation data for the common trans-Pacific coral *P. lobata*^21^. Mantel tests between the number of model larvae exchanged and the amount of genetic differentiation between each pair of populations (expressed as F'_ST_) indicated that the biophysical model explained 15% of the variation in the genetic data. This value increased to 52% when the number of modelled larvae exchanged was expressed on a logarithmic scale, a common transformation used to highlight rare long-distance connections^33^,^34^ (Supplementary Fig. 2). In comparison, Euclidian distance between reefs explained 26% of the genetic data, and 37% when converted to a log-10 scale.

We explore whether the patterns of realised connectivity seen in the genetic differentiation data for *P. lobata* are better reflected by dispersal conditions present under specific ENSO states by comparing the model–genetic correlation for the 5 representative annual periods highlighted in Fig. 2a. Correlation between the biophysical model and the genetic data was highest for the strongest 2010–2011 La Niña and weaker 2009–2010 El Niño (both 47%) followed by the weaker 2008–2009 La Niña (45%) and strongest 1997–1998 El Niño (43%) periods compared with the neutral period (2005–2006; 35%).

For the 1997–1998 El Niño period, increasing the maximum larval duration and reducing mortality rate improved the model–genetic correlation from 43 to 51% and 50%, respectively. This increase in correlation was due to a greater number of inter-regional connections being made (Supplementary Fig. 1), predominantly within the ETP, which were otherwise significantly reduced during this El Niño event relative to other periods within the decade (Fig. 3). Of these additional connections, two were new intra-regional connections not previously simulated at any time during the full study period (from PIT to EAS, and REV to COC; Fig. 3a; Supplementary Fig. 1). However, sampling gaps in the genetics data set (Supplementary Fig. 3) as well as limited distribution of *P. lobata* compared with the model coverage (*P. lobata* is replaced by *P. evermanni*, a previously unrecognized species, in the northern ETP^35^), limit the model–genetic comparison, and mean that the influence of the additional connections involving the Pitcairns, Easter and Revillagigedo Islands cannot be tested at present.

## Discussion

It has been proposed that El Niño events enhance eastward dispersal across the vast expanse of the Eastern Pacific Barrier, thereby lessening the isolation of reefs in the Eastern Pacific^3^,^14^,^19^,^20^,^23^,^24^,^25^,^26^,^27^. Following the extreme 1982–1983 El Niño, for example, the recording of a number of Indo-Pacific mollusc, echinoderm and fish colonists in the ETP was tentatively ascribed to dispersal from the central Pacific via the NECC^3^ (Fig. 1). For the modelled broadcast spawning coral, we find that, although central Pacific larvae from the NLI do reach their most easterly range of dispersal during the extreme 1997–1998 El Niño when compared with four other representative annual periods (Fig. 4c), no connections were made eastward from the central to eastern Pacific at any time over the study period (Fig. 3a). This failure to connect eastward across the EPB occurs despite maximizing potential for long-distance dispersal, and is also robust to variations in larval duration, mortality rate and numbers of larvae modelled. Significantly, this result suggests that ETP coral populations have been isolated from central Pacific sources of larval replenishment over the 14.5 years studied, implying that present ETP populations consist entirely of survivors of the extreme 1997–1998 El Niño event and recruits from local sources. This hypothesis is supported by a recent survey of a dense *Pocillopora* reef in the Galapagos^36^, which was found to consist entirely of a single clone propagated asexually from colony remnants following the 1997–1998 El Niño-related mortality event.

The modelled dispersal paths demonstrate how, due to limited area for coral settlement in the ETP, subtle details in surface flow mean crucial east-to-west connections are missed (Fig. 4). This highlights the value of biophysical dispersal modelling in providing additional detail on the complexity of surface flows not captured by generalized circulation patterns. For example, the timing of maximum eastward dispersal from the NLI (May–September 1997; Supplementary Movies 1 and 2), corresponds to the early development stage of El Niño, when easterly surface flow intensifies as the easterly trade winds weaken and westerly wind bursts develop^8^,^37^. However, modelled dispersal paths during this period pass ∼100–200 km to the south of CLI, the key stepping stone for onward connections into the ETP (Fig. 3), and fall short of reaching reefs further within the ETP (Fig. 4c,iii). A southerly shift in the position of the NECC at the longitude of CLI (∼110° W) is a persistent feature of eastern Pacific type El Niños^37^ (‘EP'-El Niño in Fig. 3a). In contrast, no enhanced eastward dispersal from the NLI occurs during the 2009–2010 El Niño (Fig. 3), instead dispersal is even more curtailed than that seen during neutral (Fig. 4) or La Niña conditions (Fig. 4 v,vi). This outcome is consistent with the observation that central Pacific El Niños (‘CP'-El Niño in Fig. 3a) have a negligible influence on the NECC, particularly in the ETP^37^.

Breaking the model dispersal paths down by release month (Supplementary Movies 1–6) shows significant seasonal variation in dispersal direction. While dispersal paths from the NLI reached their most easterly longitudes in May to October 1997, more easterly dispersal was a persistent pattern of the boreal summer in other years (May/June to August; Supplementary Movies 1 and 2). This corresponds to seasonal strengthening of the NECC, beginning in the eastern Pacific from August and propagating west across the central Pacific during October–December^38^ (note that the effect on dispersal is integrated over the 4-month larval transport period; for example, larvae released in August could be transported into November). This seasonality in the NECC intensity can also be seen in the trajectories from CLI, which extend eastward into the ETP between May and September (Supplementary Movies 5 and 6).

Conditions during the 1997–1998 El Niño event also promoted the most westward dispersal of larvae across the EPB compared with the other representative annual periods (from both the GAL and CLI from November 1997 to the end of the releases in August 1998; Fig. 2b,c,ii; Supplementary Movies 3–6) and the largest number of larvae from the ETP successfully reaching reefs in the central Pacific occurred for releases following the 1998 and to a lesser extent 2003 El Niño events (Figs 2a and 3c). However, this was not a unique situation; westward cross-EPB connections from the GAL to central Pacific were simulated in 11 out of the 14.5 years of the study (Fig. 2). Releases in the middle to latter half of the year (June/July onwards, excluding the end of the 2002–2003 El Niño in early-2003) appeared more likely to make westward cross-EPB connections (Fig. 2b, Supplementary Movies 3 and 4), possibly partially corresponding to strengthening of the SEC during the Austral winter when the southeasterly trades are at their strongest^39^. However, the dispersal paths from the GAL were extremely complex, possibly confounded by seasonal shearing between the westward-flowing SEC and eastward-flowing NECC and eddies generated by Tropical Instability Waves^40^.

These rare westward cross-EPB connections identified by the biophysical model (Fig. 2) are not supported by the only available genetic data for a broadcast spawning coral species across the region (*P. lobata*), which suggest no gene flow between the central and eastern Pacific in either direction for potentially hundreds to thousands of years^21^. There are a number of reasons why the westward connections suggested by the model may not be realised for this species. In particular, westward-EPB connections will likely be overestimated in the model because larvae were released daily all year-round, while in reality coral spawn over discrete periods that are species-dependent but at the most a few months per year^41^. As discussed above, the model demonstrates marked variability in the direction of dispersal pathways depending on the month of release. These results are intentionally unconstrained due to our aim to model the maximum possible dispersal potential for a generic, ubiquitous species (see Methods). Observations of coral spawning in the eastern Pacific are almost non-existent. However, in the case of *P. lobata*, indirect evidence from the GAL points to larval releases occurring during at least May to June^42^. Consequently, for example, the peaks in cross-EPB connections via the GAL occurring for releases from November to December 1997, July to August 1998 and March 2003 in the model (Fig. 2) would not be realised for this species.

A further consideration is that, while the biophysical model provides predictions of the pre-settlement distribution of larvae, genetic data also integrate over post-settlement processes, including mortality, predation and competition, which will reduce the population evidence of dispersal connections^43^. After the long journey across the EPB, settling larvae face very different conditions in the arrival location compared with their origin, likely resulting in post-settlement selection against migrants^44^. Of further significance is the probable impact of El Niño conditions. Widespread El Niño-related mortality in the central and eastern Pacific^9^,^10^,^11^,^12^ would open up settlement habitat for new recruits, but any migrants would then also have to survive the elevated temperatures and subsequent secondary stresses caused by the event^9^. Further, immediate and long-term reductions in coral fecundity following bleaching stress^45^, reduced adult spawning populations due to bleaching-related mortality^10^ and direct impact of elevated temperatures on larval development^46^, would significantly reduce larval dispersal potential during and following El Niño. Since reefs across the central and eastern Pacific were exposed to bleaching-level thermal stress starting in April 1997 through June 1998 (ref. 47), it is, therefore, highly unlikely that any of the modelled cross-EPB connections via the GAL archipelago or CLI were realised in 1997–1998.

Despite the caveats discussed above—the different aspects of the dispersal process captured by the two approaches (that is, differences in temporal resolution and pre- versus post-settlement) as well as lack of model parameterization for any single species, a surprisingly large amount of the variation in reported genetic differentiation between populations of *P. lobata*, up to 52%, can be explained by the modelled connections for a generic broadcast spawning coral over the 14.5-year period. This has a number of potential implications. First, that pre-settlement dispersal patterns, set primarily by the oceanographic conditions, are roughly as important in driving genetic patterns as post-settlement selection. Second, that these oceanographic controls appear to have been relatively persistent over time, considering that genetic data integrate over multiple generations (amounting to centennial-millennial timescales), whereas the model covers only a 14.5-year period. Finally, the correlation also implies that the generic model may be generally applicable to specific species. Further testing is currently limited by a lack of data (both genetics and field observations) across the region, both to compare the model with other species, or to parameterize the model for a specific species (for example, population sizes, larval characteristics and timing of spawning). This has, however, been done for coral species in other regions; *Orbicella* (previously *Montastraea*) *annularis* in the Caribbean^48^ (46% correlation) and *Acropora hyacinthus* (75–89%) and *A. digitifera* (48–83%) in Micronesia^49^.

Returning to the hypothesized importance of El Niño events for gene flow in the eastern Pacific, are the patterns of genetic connectivity for *P. lobata* better reflected by the dispersal conditions present during El Niño? Comparing the model–genetic correlation between years indicates that ENSO events of both signs do have a larger influence on the realised connectivity captured in the genetic data when compared with neutral conditions (8–12% increase in the variation in the genetic data explained). However, it is difficult to determine specifically what about the La Niña/El Niño periods compared with the neutral period, such as changes in the strength and/or number of connections within the eastern and/or central Pacific, is driving the improvement in the correlation with the genetic data. Regarding potential cross-EPB dispersal, while there is some correlation between modelled cross-EPB connections from the GAL and the genetic data (∼6% for the full model time period), the genetic data indicate that such connections were not recent, although increased samples would be needed to strengthen this conclusion. Novel genotyping-by-sequencing approaches promise to provide higher resolution of population genetic structure in non-model organisms and could yield additional insights with regards to the timing and direction of any gene flow across the EPB in reef-building corals.

Areas where the model differs from the available genetic data suggest prudent sites for further empirical research—particularly significant given the ongoing strong El Niño event in the Pacific. These areas include Clipperton, the Galapagos, Northern Line Islands, Marquesas Islands and Tuamoto, as well as locations currently missing in existing data sets such as the Revillagigedos Islands. Importantly, the model is able to suggest specific sites within these regions where connections may occur to focus field efforts. For example, connections from the GAL to MAR regions in the model occurred predominantly into the northernmost MAR Islands, which were not sampled in the genetic data (Supplementary Fig. 3; Supplementary Table 1). Further, improved modelling incorporating field and laboratory data, particularly timing of spawning, for specific species at the key locations mentioned above is required to make more accurate predictions of dispersal patterns. However, in the absence of such data, an alternative approach would be to conduct sensitivity analyses of the model output to biologically realistic variations in the timing of spawning and other biological parameters, to quantify uncertainty and guide future empirical work.

Finally, genetic data for non-coral species (for example, the gastropods *Conus ebraeus*^50^ and *C. chaldaeus*^51^, sea urchin *Echinothrix diadema*^52^, and boxfish *Ostraceon meleagris*^25^) have provided evidence for connections across the EPB, including from west to east^25^ and between Hawaii and the ETP. Our model suggests that both easterly cross-EPB dispersal, as well as connections in either direction between Hawaii and the ETP, are highly unlikely to have occurred at any time over the previous decade for surface-dwelling larvae. This does not, however, exclude this possibility for species with differing dispersal modes, such as lower buoyancy or vertical swimming behaviour and consequently a greater depth in the water column. Due to occasionally marked differences in flow with depth, especially in areas of strong surface heating, such organisms could follow different dispersal paths to those presented here. Our results also do not consider the potential for longer-distance dispersal by rafting of adult corals on drifting materials, observed for colonies at least 1 year old^53^. For example, reports of pumice arriving at Hawaii and Christmas Island (NLI) originating from the Revillagigedos Islands (Fig. 1) in the ETP^54^ suggest that westward dispersal across the EPB by rafting of adult corals on drifting material beyond the 4-month larval duration limit set in our model could be possible. Indeed, model larvae trajectories from CLI did approach within ∼200–300 km of the southern tip of Hawaii within the 120-day larval duration when mortality and larval settlement were excluded (Fig. 4b). In contrast, dispersal paths from Hawaii are swept rapidly to the west by the NEC (data not shown), excluding the possibility of eastward dispersal into the ETP via this route.

Our results demonstrate the sensitivity of dispersal patterns across the equatorial Pacific to surface oceanographic conditions. These patterns show significant complexity on intra- and inter-annual scales, indicating that variations on decadal (for example, Pacific Decadal Oscillation) and longer timescales must also be considered. Our results suggest that future changes in the frequency, intensity and dynamics of ENSO events^28^,^29^ are likely to have implications for coral connectivity, and therefore the resilience of reefs Pacific wide to climate change. However, predictions of how these changes will impact connectivity patterns based on generalized circulation patterns are too simplistic. Biophysical dispersal modelling, incorporating high-resolution oceanographic data and incorporating more detailed empirical data on a greater range of species, is required to make more accurate forecasts.

## Methods

### Biophysical dispersal model

Following the methods of Wood *et al*.^55^, we modelled the dispersal of coral larvae using the Connectivity Modelling System, an open-source program developed to model the dispersal of large numbers of biotic or abiotic particles in the marine environment^56^. The connectivity Modelling System is a stochastic Lagrangian (water parcel following) Individual Based Model (IBM), which uses inputted oceanographic data to advect and track particles in a three-dimensional ocean, incorporating individual variability in particle attributes and representation of habitat for larval release and settlement.

Model larvae were ‘released' from 636 reef release sites across the central and eastern Pacific (Fig. 1 and see ‘Biological parameterization', below) over a 14.5-year period: 1 January 1997 to 3 July 2011. This represents the longest time period of any coral dispersal modelling study to date, encompassing a decade and a half of oceanographic variability including a full spectrum of ENSO conditions; both the extreme 1997–1998 El Niño and 2010–2011 La Niña, as well as a number of smaller ENSO events and ‘neutral' and transitional periods, and includes both ‘types' of El Niño: central Pacific (2004–2005 and 2009–2010) and Eastern Pacific types (1997–1998 and 2006–2007; Fig. 2a)^32^.

The model larvae were advected using daily (at 00Z) surface ocean current data from the HYbrid Coordinate Ocean Model (HYCOM^57^; GLBu0.08/expt_19.1). At 1/12° (∼9 km), the spatial and temporal resolution of HYCOM is sufficient to capture transient features such as eddies, which may act to entrain or transport larvae in the open ocean^58^,^59^,^60^. To account for smaller-scale diffusive turbulent motion not captured at this resolution, a stochastic ‘random walk' impulse was applied to each model larva at each 4-h time step, using a horizontal diffusion coefficient of 7 m^2^ s^−1^ based on the relationship between model resolution and diffusion defined by Okubo (1971). Note that the model time period from 1 Jan 2004 to the last release on the 7 November 2011 was run using an earlier release of the HYCOM global reanalysis data than the earlier period 1 January 1997 to 31 December 2003, due to the release of new HYCOM reanalysis data extending back to 1992 mid-way through the study in late 2014. While the horizontal resolution remained constant between the 2 data sets, the depth of the surface layer over which the currents used to drive the dispersal models were averaged reduced slightly from 3 to 1 m in the newer data (see Sensitivity analysis, below).

### Biological parameterization

As the focus of this study was to test the hypothesis of increased probability of rare long-distance dispersal across the EPB during El Niño events, our approach was to maximize the potential for rare long-distance dispersal, within realistic limits of dispersal parameters reported in the literature for broadcast spawning coral species. Due to significant variability in the biological factors influencing dispersal both between and within species, as well as a lack of empirical data for parameterization across the study region, we did not attempt to recreate dispersal patterns for any specific species. As such, we do not claim to attempt to recreate biological realism in the model. Model larvae were instead parameterized to represent the positively buoyant larvae of a generic, ubiquitously distributed broadcast spawning coral with high dispersal potential.

Field observations of positively buoyant coral larvae report aggregates restricted to the top few cm's of the water column, even under moderate winds (<8 m s^−1^)^61^,^62^. It is only during strong winds that some larvae could potentially be mixed deeper into sub-surface currents (∼20 m)^62^, conditions that concurrently homogenize flow depth profiles. Model larvae were therefore restricted to the surface layer in the model (that is, the model was run in two-dimensional mode only).

Reef habitat for larval release and settlement was defined using combined global reef and non-reef coral community distribution data from ReefBase v.2000 (ref. 63) and UNEP-WCMC 2010 (ref. 64). Due to computational constraints, this combined reef data was re-gridded onto a 1/6° grid, giving 636 ∼17 × 17 km reef habitat ‘cells' (Fig. 1, and see below). A total of 1,600 model larvae (see Larval release numbers, below) were released daily from the centre of each habitat cell year-round, giving 5,054 release events and over 5.1 billion larvae modelled. While this represents a highly unrealistic spawning scenario (see Discussion), in line with our aim this allows us to capture the maximum amount of variation in dispersal paths driven by seasonal variability in ocean currents, providing an oceanographically driven ‘baseline' over which inter-species, geographical and temporal variability in spawning times will reduce the modelled dispersal estimations. As equal numbers of larvae were released from each habitat cell, larger numbers of habitat cells in areas of higher reef cover approximates for larger adult population sizes in these areas.

To obtain estimates of potential connectivity (exchange of individuals between populations via dispersal), model larvae passing within a reef cell within a prescribed ‘competency window' (see below) were considered ‘settled', their advection stopped and their source and arrival location recorded. This information was then summed for all habitat cells within each region, (see Fig. 1) to build the regional connectivity matrix, (Fig. 3). Due to the gridding method used to transform the reef distribution data into a manageable number of release sites for the model, some cells extended further from the land mask of the oceanographic data than others (due simply to how the grid lay in relation to the land mask). This meant that the distance a larva could get to the coast before being considered ‘settled' varied from cell to cell. As the habitat grid was created at half the resolution of the HYCOM fields (1/6°) and aligned to this grid, the maximum distance a habitat cell could extend from the model ‘coast' was 1/6° (∼18.5 km at the equator) and the minimum ½ of a cell or 1/12° (∼9 km at the equator). This also meant that the habitat area for settlement varied by cell and by region, with again the maximum area per cell being a whole cell (∼18.5 × 18.5 km at the equator) and the minimum being ¼ of a cell (∼9 × 9 km at the equator). This method differs from smaller-scale dispersal modelling approaches, where a uniform buffer (a ‘sensory zone' for larvae) is applied around known reef extents, and should be noted as a caveat to our results.

The inclusion of larval behaviour was not considered appropriate given the scale of this study and size of the habitat settlement cells, as coral larvae are weak swimmers and active seeking and orientation behaviour is likely to play a role at scale of metres at most^65^. However, the size of the habitat cells, while computationally driven, could be considered to compensate for near-shore processes not captured at the resolution of the oceanographic fields that may entrain larvae near coastlines^62^, maximizing the potential to capture rare connections. As we are not concerned with the actual numbers of dispersers (see ‘Larval release numbers', below), but rather relative levels of connectivity between locations and the potential for long-distance dispersal, individual particles in the model can be thought of as ‘packages' of larvae, which, if transported near enough to a reef, have at least some probability of some individuals being entrained and reaching it.

While we parameterize the model to maximize dispersal potential to capture ‘extreme' dispersal events, we incorporate basic biological parameters as well as inter-individual variability in these parameters known to be an important factor driving connectivity patterns^66^. This gives a realistic shape to the dispersal ‘kernel', whereby the majority of larvae settle close to their natal reef and a relatively small proportion are transported longer distances^67^, allowing for both local retention and rarer long-distance dispersal and giving estimates of relative levels of connectivity between locations. Following fertilization (which for broadcast spawning corals occurs in the water column), larvae must first undergo a period of development before they are able (competent) to detect and settle onto suitable reef habitat and undertake metamorphosis to the adult coral stage. To approximate variation in this ‘pre-competency' duration in the model, 10% of each release ‘cohort' became competent to settle from day 1 to 10 following release, within the range of values reported from laboratory studies^31^. Once developed, larvae have a finite amount of time over which they can survive in the plankton and still be competent to settle and complete metamorphosis into an adult coral polyp. To achieve variability in this larval duration, larvae were subjected to a constant mortality probability value of 0.02 per day, corresponding to a half-life of 35 days. This value was not based on experimental data, but rather chosen to give an exponentially decreasing number of particles competent to settle over time within our specified maximum competency duration. In this case, larvae were transported to a maximum of 120 days, the maximum competency duration reported in the literature for any broadcast spawning coral^31^, and terminated at that point if not already settled or dead. In this way, we cover both between-species (maximum possible values: 120-day larval duration, 10-day maximum pre-competency period, ubiquitous distribution) and within-species (a full range within the above) variability. Therefore, for some species, for example, short pre-competency durations of 1–2 days may overestimate the amount of local retention, while the long maximum competency of 120 days may overestimate long-distance dispersal.

Finally, an additional run was conducted excluding larval biology (mortality and settlement), in which model particles were simply advected to a maximum of 120 days, incorporating stochastic turbulent motion only, and their trajectories plotted (Fig. 4). This gives an idea of the maximum ‘oceanographic range' for the study period, excluding the influence of biology. As this run was more computationally demanding, 20 larvae were released daily from CLI (1 reef cell), the GAL (43 reef cells) and NLI (22 reef cells) over five representative annual periods (see below) only.

It must be noted that we only model ‘potential' connectivity—arrival of a larvae at a reef site does not imply successful settlement of the larvae onto the reef or recruitment into the host population. This process, which defines true population connectivity, is controlled by a host of further factors, such as predation and competition, which are not possible to include in the model.

### Inter-annual analysis

Five representative annual periods were extracted from the full model output to compare between different years experiencing different ENSO conditions (Figs 3 and 4); the extreme 1997–1998 El Niño (an eastern Pacific event^32^), a weaker, central Pacific El Niño (2009–2010), the extreme 2010–2011 La Niña, a weaker La Niña (2008–2009) and a neutral period (2005–2006). Releases from 1 June to 31 May the following year were selected, to cover a full annual cycle of seasonal variability in circulation, centred around the peak of typical El Niño/La Niña events (December) to capture both the build-up and tail of these events.

### Sensitivity analysis

Sensitivity of the model output to the maximum competency duration and mortality rate was tested over the 1997–1998 El Niño period, as the most likely period for eastward cross-Pacific dispersal. Two additional runs, attempting to further maximize the potential for long-distance dispersal, were conducted for this period; (1) 130 instead of 120-day maximum larval duration and (2) 70 instead of 35-day half-life (Supplementary Fig. 1). A total of 1,600 larvae were released daily from all 636 locations from 1 June 1997 to 31 May 1998. All other conditions remained constant.

The number of larvae released per event was primarily computationally constrained. However, in the absence of empirical data on the size of adult populations or their reproductive output across the region, biologically realistic parametrization for larval release numbers was not considered feasible or applicable for this study. Instead, sensitivity of model output on the scale of interest (inter-regional connections) to larval numbers was tested to determine the appropriate number of larval releases per site. For this, the model was run multiple times with releases from 3 November 2003 to 3 July 2011 with an increasing number of larvae per daily release (*N*); from *N*=90 to *N*=1,600, and the total number of inter-regional connections (regions defined as in Fig. 1) obtained calculated for each run (Supplementary Fig. 4). Individual runs at each value of *N* were also repeated to gain an idea of the spread in the number of connections obtained due to model stochasticity (Supplementary Table 2). At 1,600 larvae per release, the total number of connections between regions levels off at 125 (Supplementary Fig. 4), suggesting that the model system (given the 8 years of oceanographic data and biological parameters used) is ‘saturated' with respect to the number of inter-regional connections at this scale. However, the overall number of connections likely reflects a balance of both losses and gains in connectivity due to model stochasticity, that is, specific connections may vary even as the total number of connections levels off. To test whether this was the case, runs of incrementally increasing larval numbers were created by summing the output of smaller runs (Supplementary Table 2). Difference matrices between each run were then plotted, showing connections lost or gained between runs (Supplementary Fig. 5). Up to *N*=800, a small number (1–2) of inter-regional connections were gained by increasing the number of larvae modelled. At *N*>800, no differences in specific inter-regional connections occurred within the study area, although 1 connection was gained between *N*=1,400 and *N*=1,600. In summary, model stochasticity does not impact large-scale connections, but will introduce local-level variability between runs.

The implications of a slightly different depth of surface layer in the HYCOM data between the 1997 and 2003 (0–1 m, downloaded in 2014), and 2004 and 11 (0–3 m, downloaded in 2013) periods used to drive the model was tested by repeating the 2010–11 La Niña period (releases from 1 June 2010 to 31 May 2011) using both the original and new HYCOM data. The result was a small overall decrease in the number of regional connections with the 1 m compared with 3 m surface layer depth data, totalling seven losses and five gains, and all, with one exception, within the ETP (Supplementary Fig. 6). However, there was no change to the key model result of rare westward and no eastward dispersal across the EPB for this period.

### Model–genetic comparison

We quantitatively compared the model output for a generic broadcast spawning coral with recently published microsatellite genetic data across the region for the common Pacific species *P. lobata*^21^. Model connectivity output for the locations where genetic data were available (Supplementary Fig. 3) was extracted from the full matrix, giving 21 sub-regional groups (152 cells; Supplementary Table 1). Note that only the following regions used in the connectivity matrices were represented by the genetic data: NHW, EHW, NLI, MAR, CLI, GAL, COC, CRP and COL (Supplementary Fig. 3). The distribution of *P. lobata* does not extend to the northern ETP, where it is instead replaced by *P. evermanni*^35^, limiting the extent of the model domain that could be compared with genetic data.

A number of transformations were made to the model output before it could be compared with the empirical data. First, as the genetic data integrate over connections operating in both directions between two populations, they therefore give triangular ‘half' matrices of the level of differentiation between each population pair. In contrast, the model output is expressed as a two-way matrix giving the number of larvae exchanged between each pair of populations in each direction. The raw model connectivity matrix was therefore firstly converted into a directionless half matrix by summing along the diagonal, that is, summing the number of particles exchanged in both directions between each pair of populations. Values along the diagonal (retention within the release location; ‘self-seeding'), not present in the genetic data, were excluded from the analysis. The resulting ‘triangular' connectivity matrix was then normalized to the total number of particles released, effectively converting the values into a probability of successful dispersal between each pair of locations (as we consider relative levels of connectivity, absolute values are not required). Second, genetic data are expressed as the level of differentiation between populations (F'st^68^) on a scale of 0–1, with 0 being no differentiation (maximum connectivity) and 1 being maximum differentiation. Large values in the model output, conversely, correspond to high levels of connectivity. The half matrix was therefore inverted to be directly comparable with the F'st data. A Mantel test was then performed to compare the measure of standardized genetic distance between locations (F'st) with both the transformed model data and approximate Euclidian geographic distance (both raw and converted to a logarithmic scale) between each pair of locations (Supplementary Fig. 2).

### Code availability

The biophysical model presented uses the open-source Connectivity Modelling System^56^. The source code is freely available to download and customize at https://github.com/beatrixparis/connectivity-modeling-system.

### Data availability

The model output data presented in this manuscript are available from the corresponding author on request. The reef distribution data used to create the habitat cells were taken from UNEP-WCMC (2010)^64^ available at http://data.unep-wcmc.org/datasets/1, and ReefBase 2000 (ref. 63) available at http://reefgis.reefbase.org/. The oceanographic data (surface u and v current velocities) used to drive the biophysical dispersal model were downloaded from the HYCOM+NCODA Global 1/12° Reanalysis data server at https://hycom.org/dataserver/glb-reanalysis. The genetic data for *P. lobata* used in the model–genetic comparison are presented in Baums *et al*.^21^ and available on DRYAD (doi:10.5061/dryad.7gp1f).

## Additional information

**How to cite this article:** Wood, S. *et al*. El Niño and coral larval dispersal across the eastern Pacific marine barrier. *Nat. Commun.* 7:12571 doi: 10.1038/ncomms12571 (2016).

## Supplementary Material

Supplementary Information Supplementary Figures 1-6 and Supplementary Tables 1-2

Supplementary Movie 1 Monthly larval trajectories from the Northern Line Islands, January 1997 to August 1998. Density of larval paths by month of release from the Northern Line Islands (NLI; 22 release cells), plotted on a 1/6^°^ grid.

Supplementary Movie 2 Monthly larval trajectories from the Northern Line Islands, November 2003 to June 2011. Density of larval paths by month of release from the Northern Line Islands (NLI; 22 release cells), plotted on a 1/6^°^ grid.

Supplementary Movie 3 Monthly larval trajectories from the Galapagos, January 1997 to August 1998. Density of larval paths by month of release from the Galapagos Islands (GAL; 43 release cells), plotted on a 1/6^°^ grid.

Supplementary Movie 4 Monthly larval trajectories from the Galapagos, November 2003 to June 2011. Density of larval paths by month of release from the Galapagos Islands (GAL; 43 release cells), plotted on a 1/6^°^ grid.

Supplementary Movie 5 Monthly larval trajectories from Clipperton Island, January 1997 to August 1998. Density of larval paths by month of release from Clipperton Island (CLI; 1 release cell), plotted on a 1/6^°^ grid.

Supplementary Movie 6 Monthly larval trajectories from Clipperton Island, November 2003 to June 2011. Density of larval paths by month of release from Clipperton Island (CLI; 1 release cell), plotted on a 1/6^°^ grid.

## References

[b1] Couce E., Ridgwell A. & Hendy E. J. Environmental controls on the global distribution of shallow-water coral reefs. J. Biogeogr. 39, 1508–1523 (2012).

[b2] Manzello D. P. . Poorly cemented coral reefs of the eastern tropical Pacific: Possible insights into reef development in a high-CO_2_ world. Proc. Natl Acad. Sci. USA 105, 10450–10455 (2008). 18663220 10.1073/pnas.0712167105 PMC2492517

[b3] Glynn P. W. & Ault J. S. A biogeographic analysis and review of the far eastern Pacific coral reef region. Coral Reefs 19, 1–23 (2000).

[b4] Glynn P. W. & Colgan M. W. Sporadic disturbances in fluctuating coral-reef environments - El Niño and coral-reef development in the eastern Pacific. Am. Zool. 32, 707–718 (1992).

[b5] Couce E., Ridgwell A. & Hendy E. J. Future habitat suitability for coral reef ecosystems under global warming and ocean acidification. Global Change Biol. 19, 3592–3606 (2013). 10.1111/gcb.12335 PMC4028991 23893550

[b6] Brown B. E. Coral bleaching: causes and consequences. Coral Reefs 16, 129–138 (1997).

[b7] Enfield D. B. Evolution and historical perspective of the 1997-1998 El Niño-Southern Oscillation event. Bull. Mar. Sci. 69, 7–25 (2001).

[b8] McPhaden M. J. Genesis and evolution of the 1997-98 El Niño. Science 283, 950–954 (1999). 9974381 10.1126/science.283.5404.950

[b9] Glynn P. W., Mate J. L., Baker A. C. & Calderon M. O. Coral bleaching and mortality in panama and Ecuador during the 1997-1998 El Niño-Southern oscillation event: Spatial/temporal patterns and comparisons with the 1982-1983 event. Bull. Mar. Sci. 79–109 (2001).

[b10] Glynn P. W. El-Niño-associated disturbance to coral reefs and post disturbance mortality by Acanthaster-Planci. Mar. Ecol. Prog. Ser. 26, 295–300 (1985).

[b11] Vargas-Ángel B., Zapata F. A., Hernández H. & Jiménez J. M. Coral and coral reef responses to the 1997-98 El Niño event on the Pacific coast of Colombia. Bull. Mar. Sci. 69, 111–132 (2001).

[b12] Glynn P. W. in *Global Ecological Consequences of the 1982–83 El Niño-Southern Oscillation* Vol. 52 (ed. Glynn, P. W.) 55–126 (Elsevier, 1990).

[b13] Glynn P. W., Riegl B., Correa A. M. S. & Baums I. B. Rapid recovery of a coral reef at Darwin Island, Galapagos Islands. Galapagos Res. 6–13 (2009).

[b14] Guzman H. M. & Cortes J. Reef recovery 20 years after the 1982-1983 El Niño massive mortality. Mar. Biol. 151, 401–411 (2007).

[b15] Glynn P. W., Purkis S. J., Kerr J. M. & Smith T. B. Coral reef recovery in the Galápagos Islands: the northernmost islands (Darwin and Wenman). Coral Reefs 34, 421–436 (2015).

[b16] Glynn P. W. State of coral reefs in the Galápagos Islands: natural vs anthropogenic impacts. Mar. Pollut. Bull. 29, 131–140 (1994).

[b17] Graham N. A. J., Nash K. L. & Kool J. T. Coral reef recovery dynamics in a changing world. Coral Reefs 30, 283–294 (2011).

[b18] Paris-Limouzy C. B. in Encyclopedia of Modern Coral Reefs Encyclopedia of Earth Sciences Series ed. Hopley D. 881–889Springer-Verlag, (2011).

[b19] Dana T. Development of contemporary eastern pacific coral reefs. Mar. Biol. 33, 355–374 (1975).

[b20] Grigg R. W. & Hey R. Palaeoceanography of the tropical eastern Pacific-ocean. Science 255, 172–178 (1992). 17756067 10.1126/science.255.5041.172

[b21] Baums I. B., Boulay J. N., Polato N. R. & Hellberg M. E. No gene flow across the Eastern Pacific Barrier in the reef-building coral Porites lobata. Mol. Ecol. 21, 5418–5433 (2012). 22943626 10.1111/j.1365-294X.2012.05733.x

[b22] Heck K. L. & McCoy E. D. Long-distance dispersal and reef-building corals of eastern Pacific. Mar. Biol. 48, 349–356 (1978).

[b23] Richmond R. H. in Global Ecological Consequences of the 1982–83 El Niño-Southern Oscillation Vol. 52 ed. Glynn P. W. 127–140Elsevier (1990).

[b24] Victor B. C., Wellington G. M., Robertson D. R. & Ruttenberg B. I. The effect of the El Niño-Southern Oscillation event on the distribution of reef-associated labrid fishes in the eastern Pacific Ocean. Bull. Mar. Sci. 69, 279–288 (2001).

[b25] Lessios H. A. & Robertson D. R. Crossing the impassable: genetic connections in 20 reef fishes across the eastern Pacific barrier. Proc. Biol. Sci. 273, 2201–2208 (2006). 16901840 10.1098/rspb.2006.3543 PMC1635520

[b26] Glynn P. W. & Wellington G. M. Corals and Coral Reefs of the Galápagos Islands Univ. of California Press (1983).

[b27] Glynn P. W., Veron J. E. N. & Wellington G. M. Clipperton atoll (eastern Pacific): Oceanography, geomorphology, reef-building coral ecology and biogeography. Coral Reefs 15, 71–99 (1996).

[b28] Yeh S.-W. . El Niño in a changing climate. Nature 461, 511–514 (2009). 19779449 10.1038/nature08316

[b29] Cai W. . Increased frequency of extreme La Niña events under greenhouse warming. Nat. Clim. Change 5, 132–137 (2015).

[b30] Veron J. E. N., Stafford-Smith M. G., Turak E. & DeVantier L. M. Corals of the World Online, version 0.01 (Beta). Available at http://www.coralsoftheworld.org (2016).

[b31] Connolly S. R. & Baird A. H. Estimating dispersal potential for marine larvae: dynamic models applied to scleractinian corals. Ecology 91, 3572–3583 (2010). 21302829 10.1890/10-0143.1

[b32] Yu J.-Y. & Kim S. T. Identifying the types of major El Niño events since 1870. Int. J. Climatol. 33, 2105–2112 (2013).

[b33] Jensen J. L., Bohonak A. J. & Kelley S. T. Isolation by distance, web service v.3.23. BMC Genet. 6, 13 (2005). 15760479 10.1186/1471-2156-6-13 PMC1079815

[b34] Slatkin M. Isolation by distance in equilibrium and nonequilibrium populations. Evolution 47, 264–279 (1993). 10.1111/j.1558-5646.1993.tb01215.x 28568097

[b35] Boulay J. N., Hellberg M. E., Cortes J. & Baums I. B. Unrecognized coral species diversity masks differences in functional ecology. Proc. Biol. Sci. 281, 20131580 (2014). 24335977 10.1098/rspb.2013.1580 PMC3871303

[b36] Baums I. B. . Marginal coral populations: the densest known aggregation of Pocillopora in the Galápagos Archipelago is of asexual origin. Front. Mar. Sci. 1, 59 (2014).

[b37] Hsin Y.-C. & Qiu B. The impact of Eastern-Pacific versus Central-Pacific El Niños on the North Equatorial Countercurrent in the Pacific Ocean. J. Geophys. Res. 117, C11017 (2012).

[b38] Hsin Y.-C. & Qiu B. Seasonal fluctuations of the surface North Equatorial Countercurrent (NECC) across the Pacific basin. J. Geophys. Res. 117, C06001 (2012).

[b39] Tomczak M. & Godfrey J. S. Regional Oceanography: an Introduction Vol. 2 (Elsevier Science Ltd. (2003).

[b40] Willett C. S., Leben R. R. & Lavín M. F. Eddies and Tropical Instability Waves in the eastern tropical Pacific: a review. Prog. Oceanogr. 69, 218–238 (2006).

[b41] Harrison P. L. & Wallace C. C. in Ecosystems of the World ed. Dubinsky Z. 133–207Elsevier (1990).

[b42] Glynn P. W. . Reef coral reproduction in the Eastern Pacific-Costa-Rica, Panama, and Galapagos-Islands (Ecuador). 2. Poritidae. Mar. Biol. 118, 191–208 (1994).

[b43] Hellberg M. Footprints on water: the genetic wake of dispersal among reefs. Coral Reefs 26, 463–473 (2007).

[b44] Polato N. R. . Location-Specific Responses to Thermal Stress in Larvae of the Reef-Building Coral Montastraea faveolata. PLOS ONE 5, e11221 (2010). 20585643 10.1371/journal.pone.0011221 PMC2890407

[b45] Ward S., Harrison P. & Hoegh-Guldberg O. in Proceedings of the 9th International Coral Reef Symposium Vol. 2 eds Moosa M. K.. 1123–1129Ministry of Environment; Indonesian Institute of Sciences; International Society for Reef Studies (2000).

[b46] O'Connor M. . Temperature control of larval dispersal and the implications for marine ecology, evolution, and conservation. Proc. Natl Acad. Sci. USA 104, 1266–1271 (2007). 17213327 10.1073/pnas.0603422104 PMC1764863

[b47] NOAA. NOAA Coral Reef Watch. Available at http://coralreefwatch.noaa.gov/satellite/analyses_guidance/enso_bleaching_97-99_ag_20140507.php (2015).

[b48] Foster N. L. . Connectivity of Caribbean coral populations: complementary insights from empirical and modelled gene flow. Mol. Ecol. 21, 1143–1157 (2012). 22276913 10.1111/j.1365-294X.2012.05455.x

[b49] Davies S. W., Treml E. A., Kenkel C. D. & Matz M. V. Exploring the role of Micronesian islands in the maintenance of coral genetic diversity in the Pacific Ocean. Mol. Ecol. 24, 70–82 (2015). 25407355 10.1111/mec.13005

[b50] Duda T. F. & Lessios H. A. Connectivity of populations within and between major biogeographic regions of the tropical Pacific in Conus ebraeus, a widespread marine gastropod. Coral Reefs 28, 651–659 (2009).

[b51] Duda T. F. . Patterns of population structure and historical demography of *Conus* species in the Tropical Pacific. Am. Malacol. Bull. 30, 175–187 (2012).

[b52] Lessios H. A., Kessing B. D. & Robertson D. R. Massive gene flow across the world's most potent marine biogeographic barrier. Proc. Biol. Sci. 265, 583–588 (1998).

[b53] Jokiel P. Transport of reef corals into the Great Barrier Reef. Nature 347, 665–667 (1990).

[b54] Jokiel P. L. & Cox E. F. Drift pumice at Christmas Island and Hawaii: evidence of oceanic dispersal patterns. Mar. Geol. 202, 121–133 (2003).

[b55] Wood S., Paris C. B., Ridgwell A. & Hendy E. J. Modelling dispersal and connectivity of broadcast spawning corals at the global scale. Global Ecol. Biogeogr. 23, 1–11 (2014).

[b56] Paris C. B., Helgers J., van Sebille E. & Srinivasan A. Connectivity modeling system: a probabilistic modeling tool for the multi-scale tracking of biotic and abiotic variability in the ocean. Environ. Modell. Software 42, 47–54 (2013).

[b57] Chassignet E. P. . The HYCOM (HYbrid Coordinate Ocean Model) data assimilative system. J. Mar. Syst. 65, 60–83 (2007).

[b58] Limouzy-Paris C. B., Graber H. C., Jones D. L., Röpke A. W. & Richards W. J. Translocation of larval coral reef fishes via sub-mesoscale spin-off eddies from the Florida Current. Bull. Mar. Sci. 60, 966–983 (1997).

[b59] Sponaugle S., Lee T., Kourafalou V. & Pinkard D. Florida Current frontal eddies and the settlement of coral reef fishes. Limnol. Oceanogr. 50, 1033–1048 (2005).

[b60] Lee T. N., Clarke M. E., Williams E., Szmant A. F. & Berger T. Evolution of the Tortugas Gyre and its Influence on recruitment in the Florida keys. Bull. Mar. Sci. 54, 621–646 (1994).

[b61] Oliver J. K. & Willis B. L. Coral-spawn slicks in the Great Barrier Reef: preliminary observations. Mar. Biol. 94, 521–529 (1987).

[b62] Willis B. L. & Oliver J. K. Direct tracking of coral larvae - implications for dispersal studies of planktonic larvae in topographically complex environments. Ophelia 32, 145–162 (1990).

[b63] Vergara S. . ReefBase 2000: Improving Policies for Sustainable Management of Coral Reefs ICLARM (2000).

[b64] UNEP-WCMC, WorldFish Centre, WRI, TNC. Global distribution of warm-water coral reefs, compiled from multiple sources including the Millennium Coral Reef Mapping Project. Version 1.3. Includes contributions from IMaRS-USF and IRD (2005), IMaRS-USF (2005) and Spalding . (2001). Cambridge (UK). Available at http://data.unep-wcmc.org/datasets/1 (2010).

[b65] Wolanski E. & Kingsford M. J. Oceanographic and behavioural assumptions in models of the fate of coral and coral reef fish larvae. J. R. Soc. Interface 11, 12 (2014). 10.1098/rsif.2014.0209 PMC4233683 24966233

[b66] Palmer S. C. F., Coulon A. & Travis J. M. J. Inter-individual variability in dispersal behaviours impacts connectivity estimates. Oikos 123, 923–932 (2014).

[b67] Cowen R. K. & Sponaugle S. Larval Dispersal and Marine Population Connectivity. Ann. Rev. Mar. Sci 1, 443–466 (2009). 10.1146/annurev.marine.010908.163757 21141044

[b68] Meirmans P. G. Using the AMOVA framework to estimate a standardized genetic differentiation measure. Evolution 60, 2399–2402 (2006). 17236430

[b69] Smith C. A. & Sardeshmukh P. D. The effect of ENSO on the intraseasonal variance of surface temperatures in winter. Int. J. Climatol. 20, 1543–1557 (2000).

[b70] McPhaden M. J., Lee T. & McClurg D. El Niño and its relationship to changing background conditions in the tropical Pacific Ocean. Geophys. Res. Lett. 38, L15709 (2011).

