# Supplementary material for: El Niño and coral larval dispersal across the eastern Pacific marine barrier
Source: Nat Commun. 2016 Aug 23;7:12571. doi: 10.1038/ncomms12571 (PMC4996977; doi:10.1038/ncomms12571)
Supplement: Supplementary Information — Supplementary Figures 1-6 and Supplementary Tables 1-2 [file ncomms12571-s1.pdf]

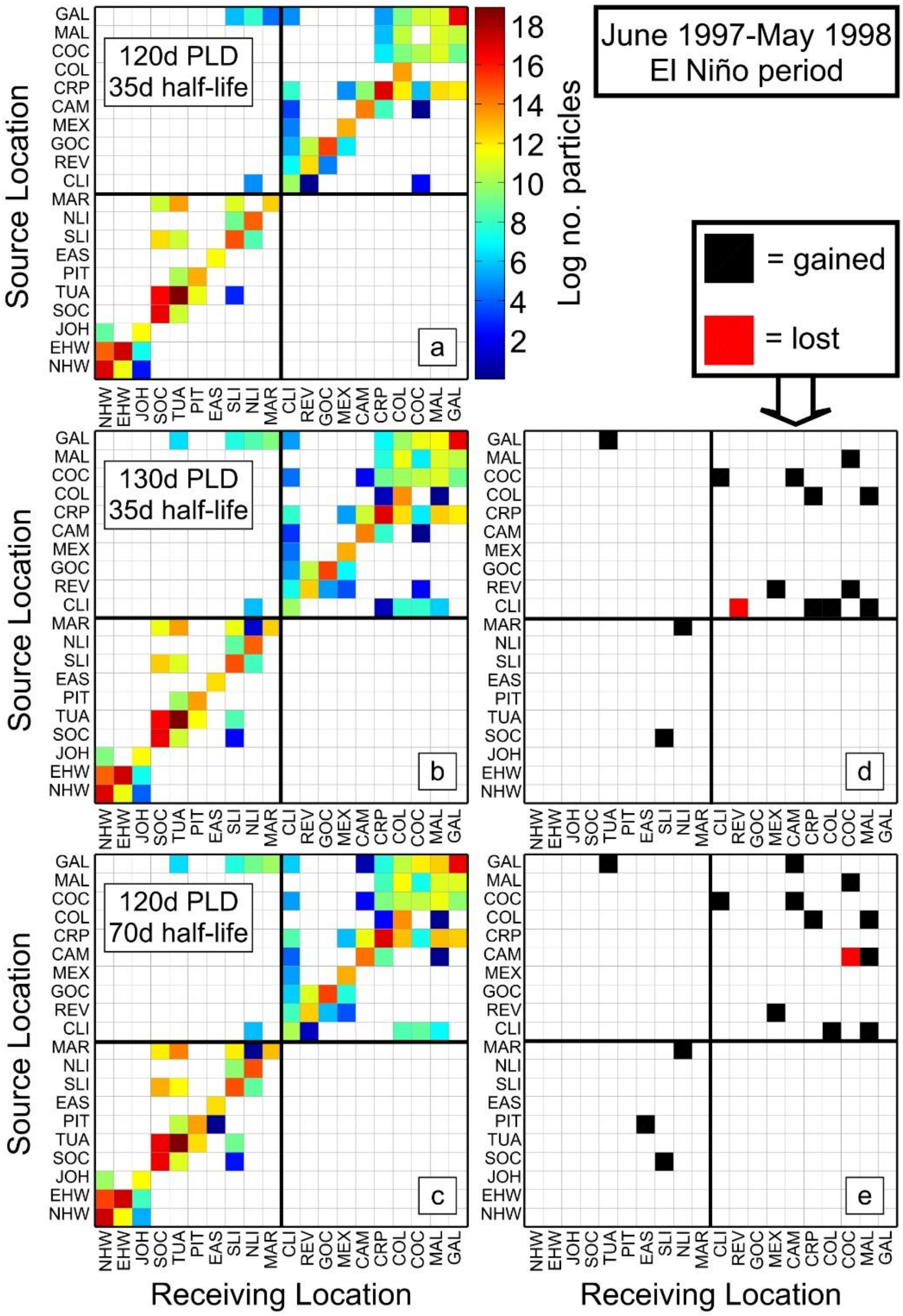

Supplementary Figure 1 | Model sensitivity to mortality and larval duration. Connectivity matrices for the 1997-98 representative El Niño year (releases from 1st June 1997 to 31st May) for **(a)** the original model: 120 day maximum pelagic larval duration (PLD) and 35 day half-life, corresponding to a mortality rate of 0.2 d^{-1} (as Fig. 3c), compared to **(b)** maximum PLD extended to 130 days (mortality rate as in **a**) and **(c)** half-life extended to 70d (mortality rate 0.01 d^{-1}, maximum PLD as in **a**). Plots **(d)** and **(e)** show regional (Fig. 1b) connections lost (red) or gained (black) in the extended PLD **(d)** and reduced mortality **(e)** runs compared to the original run **(a)**.

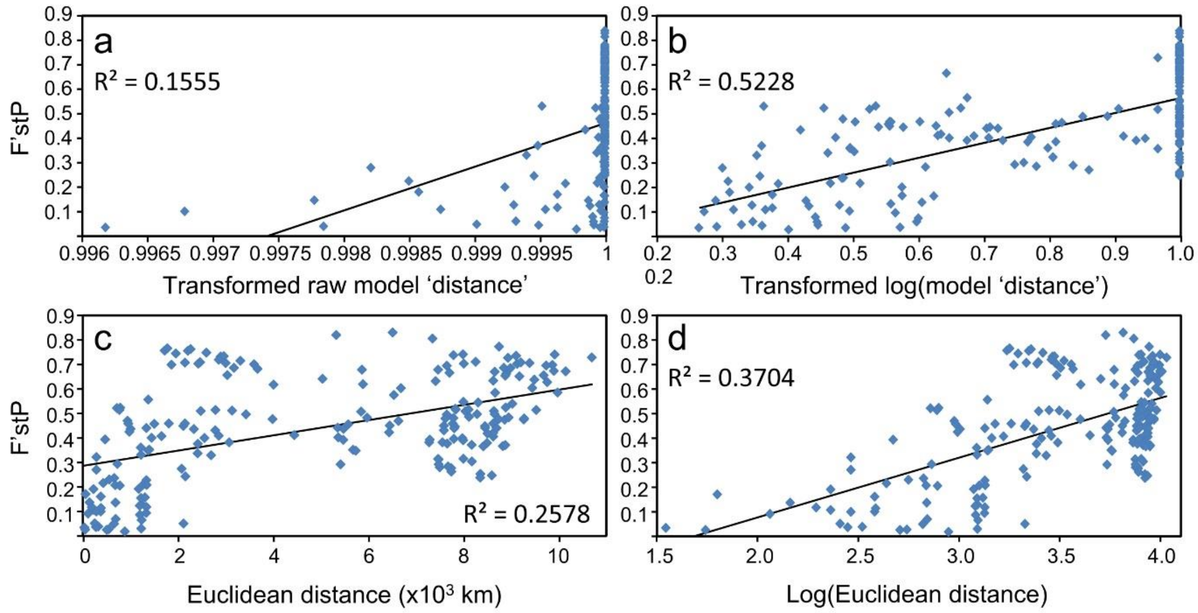

Supplementary Figure 2 | Model-genetic comparison. Mantel test results for the **(a)** raw and **(b)** log model output (i.e. 'biophysical distance') for the full 1997-2011 model run (see Methods for details of transformation), and **(c)** raw and **(d)** log Euclidean distance versus the measure of genetic differentiation (F'stP) for samples of *P. lobata* for the locations shown in Supplementary Figure 3 and Supplementary Table 1. P = 0.01.

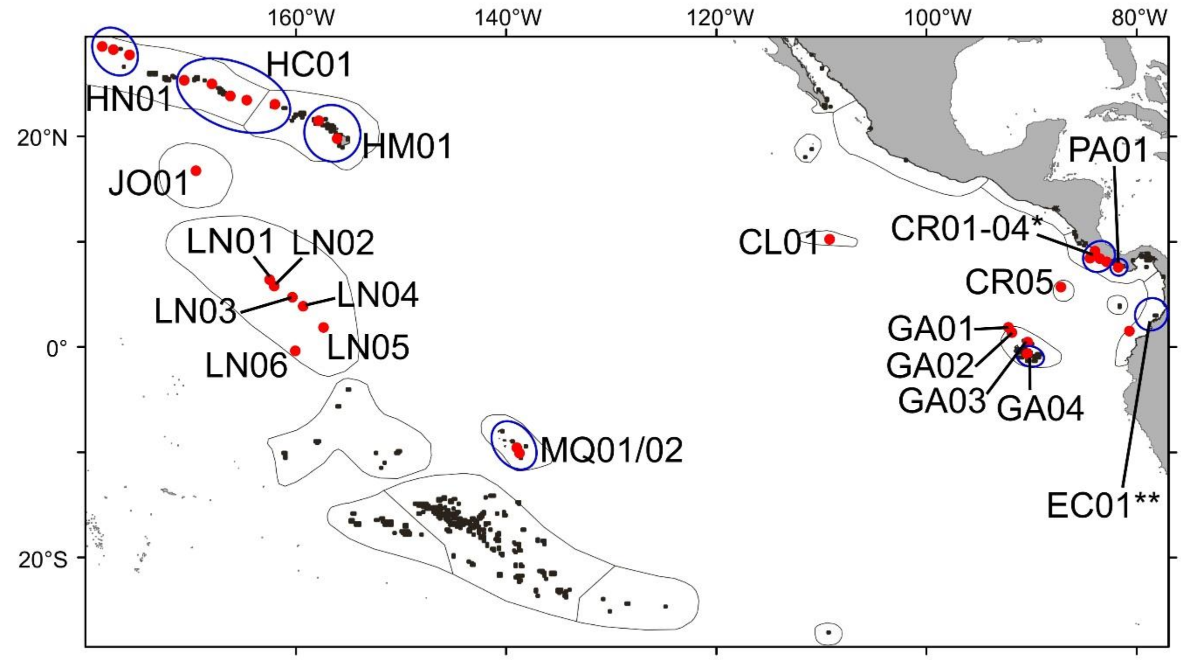

Supplementary Figure 3 | Genetic sampling locations. Locations for which genetic data for *Porites lobata*, used in the model-genetic comparison, were available (red circles). Codes correspond to the locations given in Supplementary Table 1. The blue circles indicate where output from multiple model habitat cells was combined to correspond to the appropriate sampling location, with the exception of the area marked as CR01-04* which has simply been pooled for ease of viewing. For the Hawaiian region, multiple genetic sampling sites were combined to create 3 regions (HM01, HC01 and HN01). The 2 sampling sites in the Marquesas (MQ01/02) were also combined for the analysis. **There was no habitat cell corresponding to the EC01 sampling location, therefore the nearest cell (in fact the only one on the Ecuadorian coast) was used. The thin grey lines show the outlines of the region divisions used in the connectivity matrices (Fig. 1b; i.e. only the NHW, EHW, NLI, MAR, CLI, GAL, COC, CRP and COL regions contain corresponding genetic data used in the comparison).

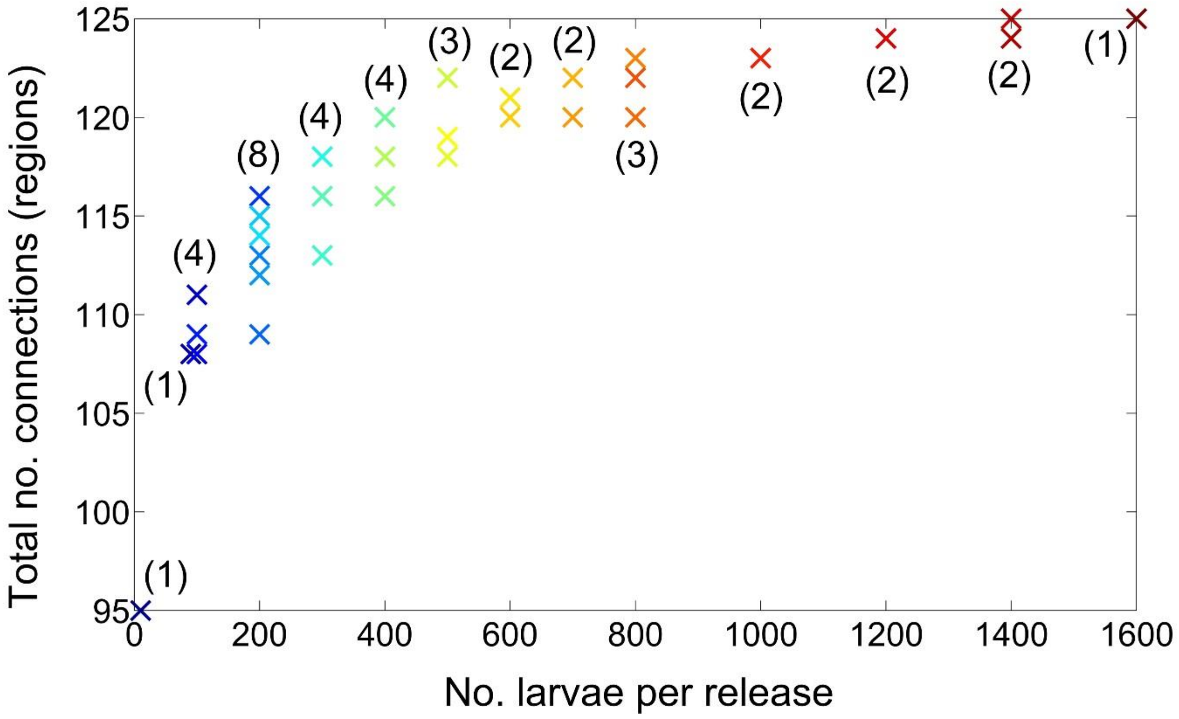

Supplementary Figure 4 | Model sensitivity to larval numbers. Total number of between-region (see Fig. 1b) connections obtained for increasing numbers of larvae per release (N) for 2004-2011 (excluding the earlier model period). Points along the same value of N (x axis) show independent runs (i) of the same number of releases in order to demonstrate within-model variability (values of i given in brackets on plot), although note that from N=800 and upwards not all runs are independent (see Supplementary Table 2 for further details).

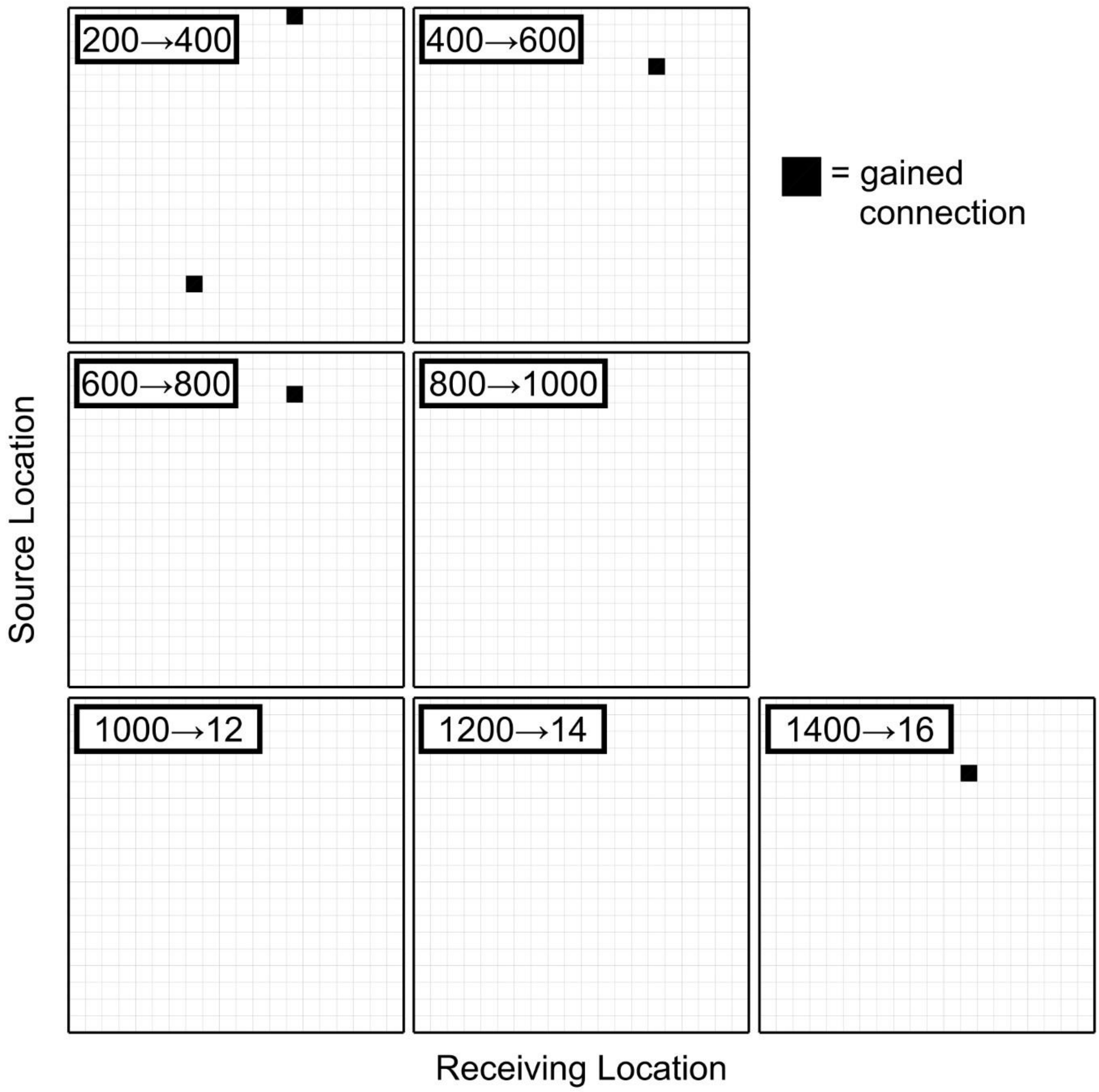

Supplementary Figure 5 | Model sensitivity to larval numbers 2. Inter-regional (Fig. 1b) connections gained (black) between runs conducted with incrementally increasing numbers of larval releases per release (200 larvae per increase, numbers given in white boxes), for 2004-2011 (excluding the earlier model period).

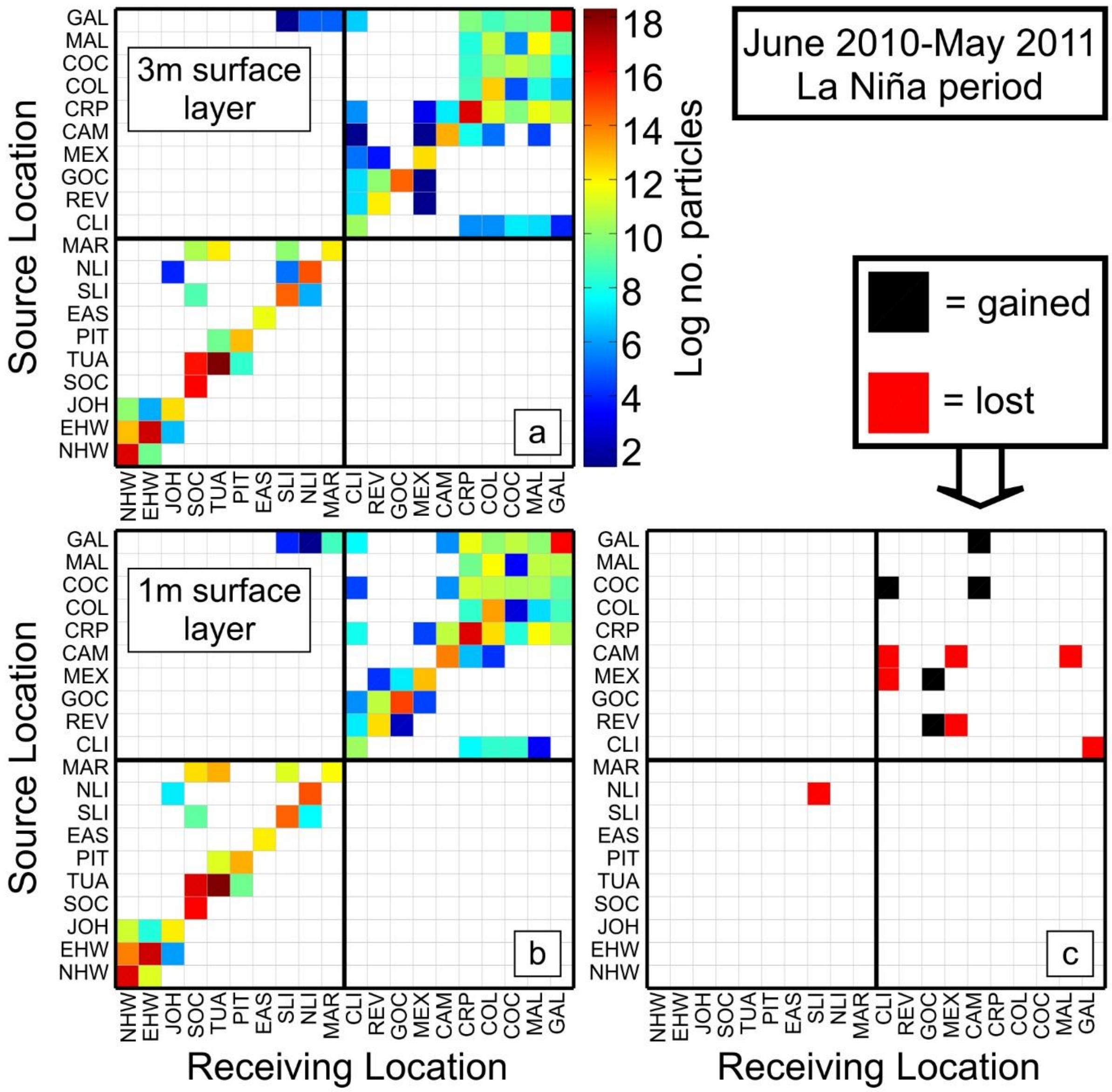

Supplementary Figure 6 | Model sensitivity to depth of surface layer. Connectivity matrices for the 2010-11 representative annual period (releases from 1st June 2010 to 31st May 2011) for **(a)** the original HYCOM data with a 3m surface layer, used for the 2004-11 model period, and **(b)** the newer date, in which the surface layer is 1m, used for the earlier 1997-2003 period. **(c)** shows differences in regional connections (Fig. 1b; red = lost connection, black = gained connection) between the two runs.

**Region**	**Code**	**Group**	**Reefs**
Hawaii	HN01	Hawaii North	Midway, Kure, Pearl & Hermes
	HC01	Hawaii Central	Maro, Necker, French Frigate Shoals, Gardner Pinnacles, Nihoa
	HM01	Hawaii Main	Oahu and Hawaii
Johnston	JO01	Johnston Atoll	
Line Islands	LN01	Kingman Reef	
	LN02	Palmyra	
	LN03	Teraina	
	LN04	Tabuaeran	
	LN05	Christmas	
	LN06	Jarvis	
Marquesas	MQ01/02		Fatu Hiva, Tahuata/**Motane**/**Hiva Oa**, Nuku Hiva/Ua Huka, Motu One *
Clipperton	CL01		
Galapagos	GA01	Darwin	
	GA02	Wolf	
	GA03	NW Islands	Marchena, Genovesa, Pinta
	GA04	Southern Galapagos	Floreana, Espanola, S. Cristabal, S. Cruz, south and east Isabela, Santiago
Costa Rica	CR01	Marino Ballena	
	CR02/03	Caño Is./Drake Bay	
	CR04	Golfo Dulce	
	CR05	Is. Cocos	

Panama	PA01		Uvas, Coiba, Gulfo de Chuiriqui,
Ecuador	EC01	La Llorona**	

Supplementary Table 1 | Locations used in the model/genetic data comparison. See also Supplementary Figure 3. *Whilst there were only 2 genetic sampling locations in the Marquesas (in bold), the entire island chain in the model was included in the analysis. ***Sampling site does not match model habitat, used nearest cells (see Supplementary Figure 3)

	i	Combination	Full combination breakdown
**10**			
**90**			
**100**	**A**		
	**B**		
	**C**		
	**D**	10 + 90	
**200**	**A**		
	**B**		
	**C**		
	**D**		
	**E**		
	**F**		
	**G**	100A + 100B	
	**H**	100C + 100D	100C + 10 + 90
**300**	**A**	200A + 100A	
	**B**	200B + 100B	

	C	200C + 100C	
	D	200D + 100D	200D + 10 + 90
400	A	200A + 200B	
	B	200C + 200D	
	C	200E + 200F	
	D	200G + 200H	100A + 100B + 100C + 10 + 90
500	A	400A + 100A	200A + 200B + 100A
	B	400B + 100B	200C + 200D + 100B
	C	400C + 100C	200E + 200F + 100C
600	A	400A + 200C	200A + 200B + 200C
	B	400C + 200D	200D + 200E + 200F
700	A	600A + 100A	200A + 200B + 200C + 100A
	B	600B + 100B	200D + 200E + 200F + 100B
800	A	700A + 100C	200A + 200B + 200C + 100A + 100C
	B	700B + 100D	200D + 200E + 200F + 100B + 10 + 90
	C	400A + 400B	200A + 200B + 200C + 200D
1000	A	500A + 500B	200A + 200B + 200C + 200D + 100A + 100B

	**B**	800C + 200E	200A + 200B + 200C + 200D + 200E
**1200**	**A**	600A + 600B	200A + 200B + 200C + 200D + 200E + 200F
	**B**	800C + 400D	200A + 200B + 200C + 200D + 100A + 100B + 100C + 10 + 90
**1400**	**A**	700A + 700B	200A + 200B + 200C + 200D + 200E + 200F + 100A + 100B
	**B**	1200B + 200F	200A + 200B + 200C + 200D + 200F + 100A + 100B + 100C + 10 + 90
**1600**	**A**	800A + 800B	200A + 200B + 200C + 200D + 200E + 200F + 100A + 100B + 100C + 10 + 90

Supplementary Table 2 | Breakdown of runs for release number sensitivity analysis. For each subset of the number of larvae per release (N), a number of different runs were conducted (i). Those in black are original runs, while those in red were built up by summing the output of the smaller runs (columns 2 and 3) into independent larger runs (i.e. with no overlap of output, plotted in Supplementary Figure 4). Runs highlighted in blue from N=800 and upwards are comprised of progressively increasing numbers of larval releases (i.e. non-independent, plotted in Supplementary Figure 5).
